# Enhanced efficacy of sequential administration of fosfomycin and linezolid against *methicillin-resistant Staphylococcus aureus*

**DOI:** 10.3389/fmicb.2025.1511707

**Published:** 2025-03-17

**Authors:** Zaixing Chen, Qin Ai, Shuai Zheng, Ziyan Chen, Sailan Wang, Na Zhang, Huiping Liu, Yanyan Liu, Jiabin Li, Xiaohui Huang

**Affiliations:** ^1^Department of Basic and Clinical Pharmacology, School of Pharmacy, Anhui Medical University, Hefei, China; ^2^Anhui Province Key Laboratory of Major Autoimmune Diseases, Anhui Institute of Innovative Drugs, School of Pharmacy, Anhui Medical University, Hefei, China; ^3^Department of Infectious Diseases & Anhui Center for Surveillance of Bacterial Resistance, The First Affiliated Hospital of Anhui Medical University, Hefei, China; ^4^Anhui Province Key Laboratory of Infectious Diseases & Institute of Bacterial Resistance, Anhui Medical University, Hefei, China

**Keywords:** linezolid, fosfomycin, sequential administration, MRSA, PK/PD

## Abstract

The aim of this study was to assess the superiority of sequential administration of fosfomycin and linezolid in combination on the efficacy of *methicillin-resistant Staphylococcus aureus (MRSA)*. The antimicrobial activity was assessed using static and dynamic bactericidal assays, along with pharmacokinetics/pharmacodynamics *in vitro* simulation models. Transmission electron microscopy (TEM) was employed to observe ultrastructural changes in *MRSA* cell walls following both sequential and concomitant dosing strategies. The results indicated that in the static time-kill assay, at MIC levels (fosfomycin at 4–8 mg/L and linezolid at 2–4 mg/L), the combination effectively inhibited *MRSA* growth under both concurrent and sequential administration; however, the sequential dosing regimen exhibited significantly greater bactericidal activity. Similarly, in the dynamic sterilization test conducted at clinically relevant doses (linezolid 600 mg and fosfomycin 2 g), a comparable trend was observed, further supporting the superior efficacy of sequential administration. TEM analysis further revealed that sequential dosing caused more extensive damage to the bacterial cell wall and nucleus compared to concomitant administration. These findings suggest that sequential administration of fosfomycin and linezolid enhances *in vitro* efficacy against *MRSA* and may provide an improved approach for managing complicated and drug-resistant infections.

## 1 Introduction

*Staphylococcus aureus* (*S. aureus*) is a major human pathogen, known for its wide array of virulence factors and its ability to develop resistance to multiple antibiotics (Lakhundi and Zhang, 2018). *Methicillin-resistant Staphylococcus aureus (MRSA)* poses a significant therapeutic challenge due to its increased resistance and worse prognosis compared to methicillin-sensitive *S. aureus* (David and Daum, 2010; Prina et al., 2015).

Linezolid, an FDA-approved oxazolidinone antibiotic, is commonly used for the treatment of *MRSA* infections. It works by binding to the 50S subunit of the bacterial ribosome, thereby inhibiting protein synthesis (Chen et al., 2020). Although linezolid has been reported to be more effective than vancomycin in treating *MRSA* infections (Kato et al., 2021), its use as monotherapy in the treatment of complicated infections does not always yield satisfactory clinical outcomes. For instance, treatment failure has been observed in critically ill patients receiving standard linezolid regimens, even when the infecting strains are susceptible to linezolid (Hemapanpairoa et al., 2019). To mitigate treatment failures and address antibiotic resistance, combination therapy has emerged as a promising strategy. Fosfomycin, an older bactericidal agent, exerts broad-spectrum activity by inhibiting bacterial cell wall synthesis (Dijkmans et al., 2017). Previous studies have demonstrated that fosfomycin, at concentrations ranging from 1 to 256 mg/L, and linezolid, at concentrations ranging from 1 to 8 mg/L, effectively inhibit the growth of various *MRSA* isolates (Valderrama et al., 2020). To date, there have been many investigations showing that fosfomycin exhibits synergistic activity with multiple antibiotics, including linezolid, resulting in a markedly enhanced bactericidal effect when the two agents are co-administered compared to the use of either agent alone (Sahuquillo Arce et al., 2006; Saravolatz and Pawlak, 2022).

The superiority of sequential antibiotic administration over concomitant administration has been demonstrated in several studies. For instance, *Staphylococcus aureus* resistant to daptomycin has been shown to regain susceptibility when pre-treated with *β*-lactams (Lew et al., 2022). Time-kill curve analyses have revealed that sequential administration of antibiotics often results in greater bactericidal activity compared to simultaneous administration. Additionally, alternating antibiotics has been suggested as a strategy to slow down the development of bacterial resistance (Batra et al., 2021; Fuentes-Hernandez et al., 2015).

While the experiment was in progress, our group observed that although the combination of fosfomycin and linezolid produced a durable bactericidal effect, fosfomycin alone had better early bactericidal activity. This observation is consistent with previous studies (Grif et al., 2001; Chai et al., 2016), suggesting that synergistic drug combinations in the treatment of severe and complicated infections may not always achieve the desired outcomes and could even lead to therapeutic failure. Based on earlier findings (Lew et al., 2022; Batra et al., 2021), it is hypothesized that sequential administration could enhance the synergistic effect of fosfomycin and linezolid, potentially by allowing fosfomycin to first disrupt the bacterial cell wall, thereby facilitating more efficient penetration of linezolid into the bacterial cells.

To optimize clinical microbiological outcomes while minimizing the risk of toxicity, pharmacokinetic/pharmacodynamic (PK/PD) modeling is a valuable tool for dose regimen decision-making (Rodríguez-Gascón et al., 2021). Previous PK/PD studies on fosfomycin and linezolid have provided insight into their pharmacokinetics (Mao et al., 2021). In this study, we modified the administration strategy to sequential dosing and demonstrated that the bactericidal effect of sequential administration was superior to that of concomitant administration in all evaluated parameters.

The aim of this study was to compare the *in vitro* antimicrobial efficacy of sequential administration of fosfomycin with conventional co-administration against *MRSA* infections. Sequential administration demonstrated superior bactericidal efficacy in vitro, providing preliminary evidence for its potential clinical application and offering new insights into optimizing *MRSA* treatment strategies.

## 2 Materials and methods

### 2.1 Bacterial isolates

A total of three MRSA blood isolates and the methicillin-resistant *Staphylococcus aureus* strain *ATCC 43300* were obtained from the Bacterial Resistance Centre of the First Affiliated Hospital of Anhui Medical University. The isolates were identified using the automated VITEK-2 system (bioMérieux, Marcy l’Etoile, France) and confirmed by a rapid latex agglutination test.

### 2.2 Antimicrobial agents and medium

Linezolid and fosfomycin were acquired from the China Food and Drug Administration (Beijing, China). All experiments utilized Mueller-Hinton broth supplemented with calcium and magnesium (CAMHB, Oxoid, UK; 25.0 mg/L Ca^2+^, 12.5 mg/L Mg^2+^) as well as Mueller-Hinton agar (MHA, Oxoid, UK). The media containing fosfomycin also included 25 mg/L of glucose-6-phosphate (Sigma-Aldrich).

### 2.3 Determination of antimicrobial susceptibility testing

Minimum inhibitory concentrations (MICs) of linezolid were assessed using the broth microdilution technique. Cultures were incubated to logarithmic phase (~1.5 × 10^8^ CFU/mL) and then diluted 150-fold to inoculate a 96-well plate with two-fold serial dilutions of linezolid. After 24 h of incubation at 37°C, the lowest antibiotic concentration with no visible bacterial growth was determined as the MIC. The MIC of fosfomycin was determined by the agar dilution method using MHA plates containing two-fold dilutions of fosfomycin, supplemented with 25 μg/mL glucose-6-phosphate. MIC results were interpreted according to the Clinical and Laboratory Standards Institute (CLSI) breakpoints for antimicrobial susceptibility testing, with *ATCC 43300* used as the quality control strain. All experiments were performed in triplicate.

### 2.4 Checkerboard assays

Checkerboard assay was used for the synergy testing. The two drugs were diluted with Mueller-Hinton Broth into a series of concentrations based on the MICs for each tested isolate. In brief, linezolid ranging between 1/64 × MIC and 2 × MIC was dispensed in each column. Then, fosfomycin supplemented with 25 mg/L of glucose-6-phosphate ranging from 1/64 × MIC to 2 × MIC was added in every row. Then, each well was inoculated with an equal volume of 1 × 10^6^ CFU/mL bacterial suspension. Plates were incubated at 37°C for 24 h and visually inspected for turbidity to determine the growth. All the experiments were performed in triplicate.

Synergy was evaluated by the fractional inhibitory concentration index (FICI): FICI = (MIC of drug A in combination/MIC of drug A alone) + (MIC of drug B in combination/MIC of drug B alone). The FICI value was interpreted as follows: FICI ≤0.5, synergy; 0.5 < FICI ≤1, additivity; 1 < FICI ≤4, indifference; FICI >4, antagonism.

### 2.5 Static time-kill assay

The *in vitro* bactericidal activity of concurrent and sequential administration of fosfomycin and linezolid was evaluated using a static time-kill assay. Bacterial suspensions in the exponential growth phase were diluted to approximately 1.0 × 10^6^ CFU/mL. The experimental groups consisted of the following: (1) Simultaneous drug administration group: Fosfomycin (FOS) and linezolid (LZD) were added to the culture system simultaneously; (2) Sequential drug administration group: FOS was added at the initial time point, and LZD was introduced after intervals of 2, 4, 6, and 8 h; (3) No-drug growth control group: Contained only the bacterial suspension. The bacterial and drug mixtures (final volume 10 mL, bacterial concentration 1.0 × 10^6^ CFU/mL) were incubated dynamically at 37°C in a constant temperature shaker. At time points 0, 4, 8, 12, and 24 h, 100 μL samples were aseptically collected. These samples were then serially diluted using 0.9% sterile saline, and 10 μL of each dilution was plated on Mueller-Hinton agar plates. After drying at room temperature, the plates were incubated at 37°C for 24 h.

### 2.6 *In vitro* PK/PD model

#### 2.6.1 Components of an *in vitro* PK/PD simulation device

**Figure 1:**
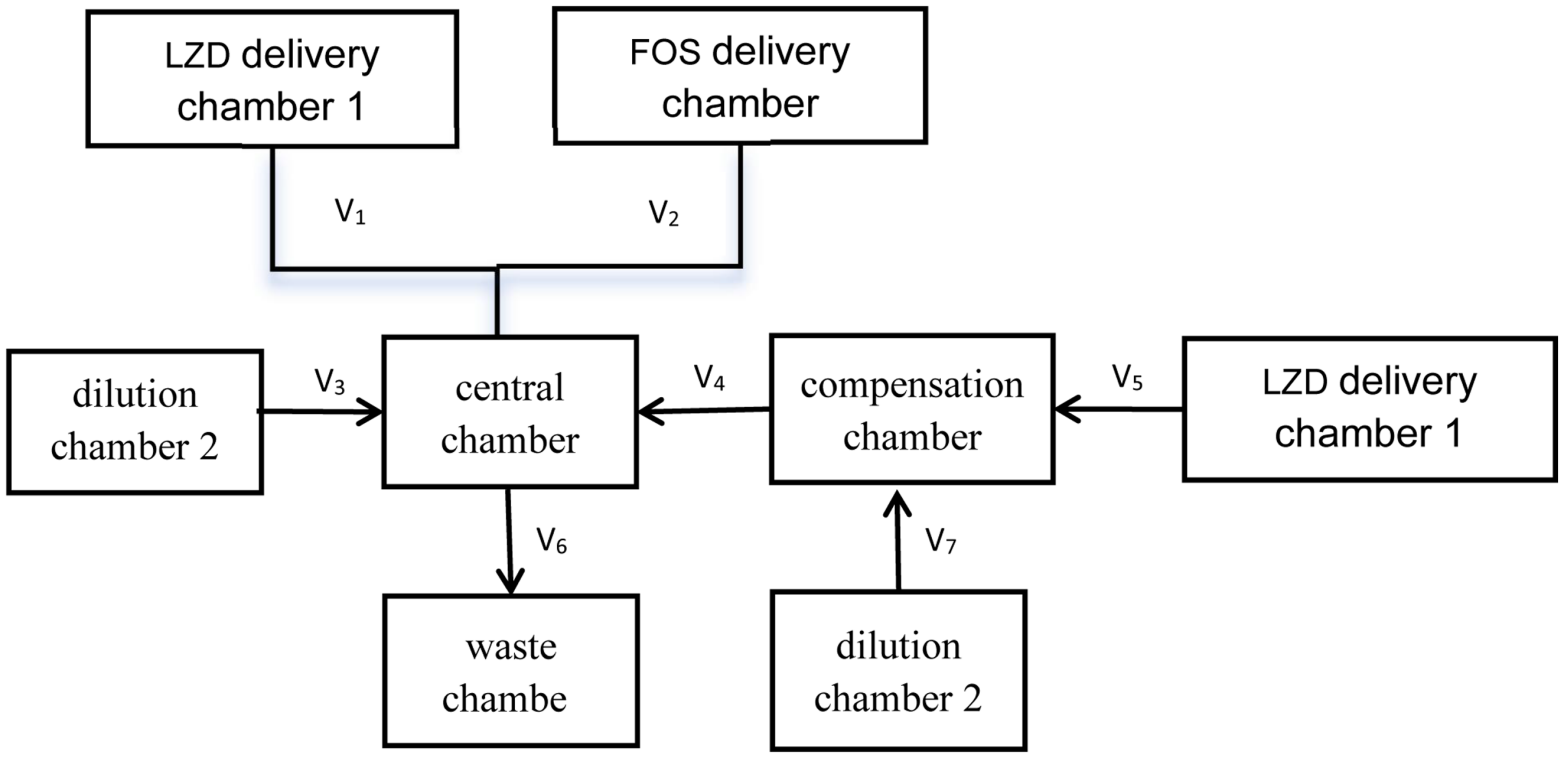
Schematic diagram of in vitro PK/PD modeling device.

An in vitro PK/PD model was constructed based on previous reports (Mao et al., 2021) to simulate the pharmacokinetics/pharmacodynamics of linezolid and fosfomycin. The device comprises a central chamber, a dilution chamber, a compensation chamber, a waste chamber, and a drug delivery chamber. The central chamber mimics the human blood circulation system, maintaining a constant volume while housing the bacterial suspension and drug mixture. Agitation ensures uniform distribution of the drug. The compensation chamber compensates for variations caused by differing drug half-lives. The dilution chamber simulates the drug clearance process by using a peristaltic pump to inject CAMHB into the central chamber while simultaneously pumping out liquid at the same rate. The pumping rate is adjusted according to the drug’s half-life to accurately replicate drug clearance. The drug delivery chamber stores linezolid or fosfomycin solutions and administers the drug to the central chamber according to a pre-programmed schedule. The waste chamber collects the effluent from the central chamber, and is connected via a 0.22 μL microporous membrane to ensure that bacteria are not directed into the waste chamber. The *in vitro* simulation device static drip process is shown in Figure 1.

$V_{1}+V_{2}+V_{3}=V_{d}\times K_{\mathrm{eLZD}}$

$V_{5}=V_{7}=V_{4}=V_{d}\times \left(K_{\mathrm{eFOS}}-K_{\mathrm{eLZD}}\right)$

$V_{6}=V_{d}\times K_{\mathrm{eFOS}}$

$V_{b}=V_{d}\times \left(K_{\mathrm{eFOS}}-K_{\mathrm{eLZD}}\right)/K_{\mathrm{eLZD}}$

$C_{\mathrm{LZD1}}=C_{\mathrm{maxLZD}}\times K_{\mathrm{eLZD}}\times V_{d}/\left(1-e^{-\mathrm{KeLZD}\cdot \mathrm{TmaxLZD}}\right)/V_{1}$

$C_{\mathrm{LZD2}}=C_{\mathrm{LZD1}}\times V_{2}/V_{5}\times V_{b}/V_{d}$

$C_{FOS}=C_{\mathrm{maxFOS}}\times K_{\mathrm{eFOS}}\times V_{d}/\left(1-e^{-\mathrm{KeFOS}\cdot \mathrm{TmaxFOS}}\right)/V_{2}$

V_1_-V_7_ represent the flow rates controlled by a computerized peristaltic pump; V_d_ is the volume of the central chamber (0.2 L); V_b_ is the volume of the compensation chamber (0.28 L); t_1/2_ denotes the half-life of the drug; K_e_ is the elimination rate of linezolid and fosfomycin; C_maxLZD_ is the maximum concentration of linezolid in the central chamber; T_maxLZD_ is the time of administration corresponding to the maximum concentration of linezolid in the central chamber; CmaxFOS is the maximum concentration of fosfomycin in the central compartment; and T_maxFOS_ refers to the time of administration corresponding to the maximum concentration of fosfomycin in the central compartment.

#### 2.6.2 *In vitro* PK/PD study

The drug delivery volumes and flow rates for each drug in the drug delivery chamber, central chamber, dilution chamber, and compensation chamber were calculated based on the above equation. The dosing regimen for the *in vitro* PK/PD device was designed as follows: for the simultaneous dosing group, fosfomycin (2 g q8h) and linezolid (600 mg q12h) were administered together; for the sequential dosing group, fosfomycin (2 g q24h) and linezolid (600 mg q24h) were administered sequentially. In the concurrent group, both drugs were infused simultaneously, while in the sequential group, fosfomycin was infused first, followed by linezolid at intervals of 2, 4, 6, and 8 h. Samples were collected and bacterial counts were performed.

#### 2.6.3 Drug concentration validation

Validation of Drug Concentrations in the *In Vitro* PK/PD Model Linezolid concentrations were determined using an HPLC-UV method based on previously established protocols (Yang et al., 2021). Fosfomycin concentrations were measured using a bioassay with *Escherichia coli ATCC 25922* as the indicator strain (Wang et al., 2021). Drug concentrations were analyzed using Phoenix WinNonlin V8.1 to compare observed pharmacokinetic parameters (C_max_, T_1/2_, AUC_0-24h_) with predicted values.

### 2.7 Characterization of cell morphology

Transmission electron microscopy (TEM) was used to observe the effects of simultaneous and sequential administration of fosfomycin and linezolid on the bacterial cell wall. The most synergistic bacterial strains were selected for the experiment. An overnight culture of *MRSA* was adjusted to a concentration of 1 × 10^8^ CFU/mL. Linezolid was used at a concentration of 4 mg/L and fosfomycin at 8 mg/L. The simultaneous administration group was incubated for 4 h, while the sequential administration group received fosfomycin first, followed by linezolid after 2 h, with a total incubation time of 4 h. Samples were centrifuged three times at 3300 rpm for 10 min at 4°C. The bacterial pellets were washed with 1 mL of phosphate-buffered saline (PBS) and fixed in 2.5% glutaraldehyde at 4°C overnight. After fixation, the samples were centrifuged and washed three times with PBS, then dehydrated through a graded ethanol series (30, 50, 70, 80, 90, and 100%). Finally, the bacterial pellets were washed twice with 100% ethanol, resuspended in ethanol, and visualized using TEM.

### 2.8 Statistical analysis

Statistical analysis was performed with SPSS 16.0. One-way ANOVA was performed to assess the change of each antibiotic concentration, alone or in combination. In the results, *p* < 0.05 was considered to be significant.

## 3 Results

### 3.1 Antimicrobial susceptibility testing

The results of the *in vitro* drug sensitivity test and fractional inhibitory concentration index (FICI) are presented in Table 1. The MICs of linezolid against the three clinical isolates were 2 mg/L, 4 mg/L, and 4 mg/L, respectively, while the MICs of fosfomycin were 4 mg/L, 4 mg/L, and 8 mg/L. The FICI values for the combination of fosfomycin and linezolid against the three strains were 0.5, 0.5, and 1, indicating either synergistic or additive effects.

**Table 1:** MIC and FICI values for MRSA strains treated with fosfomycin and linezolid.

Isolates	MIC_LZD_ (mg/L)	MIC_FOS_ (mg/L)	FICI	MIC_LZD_ (mg/L) of checkerboard assays	MIC_FOS_(mg/L) of checkerboard assays
*ATCC43300*	2	4	0.5	0.5	1
Isolate 1	2	4	0.5	0.5	1
Isolate 2	4	8	0.5	1	2
Isolate 3	4	8	1	2	4

LZD: linezolid; FOS: fosfomycin; MIC, minimum inhibitory concentration; FICI: Fractional Inhibitory Concentration Index.

### 3.2 Time-kill studies

**Figure 2:**
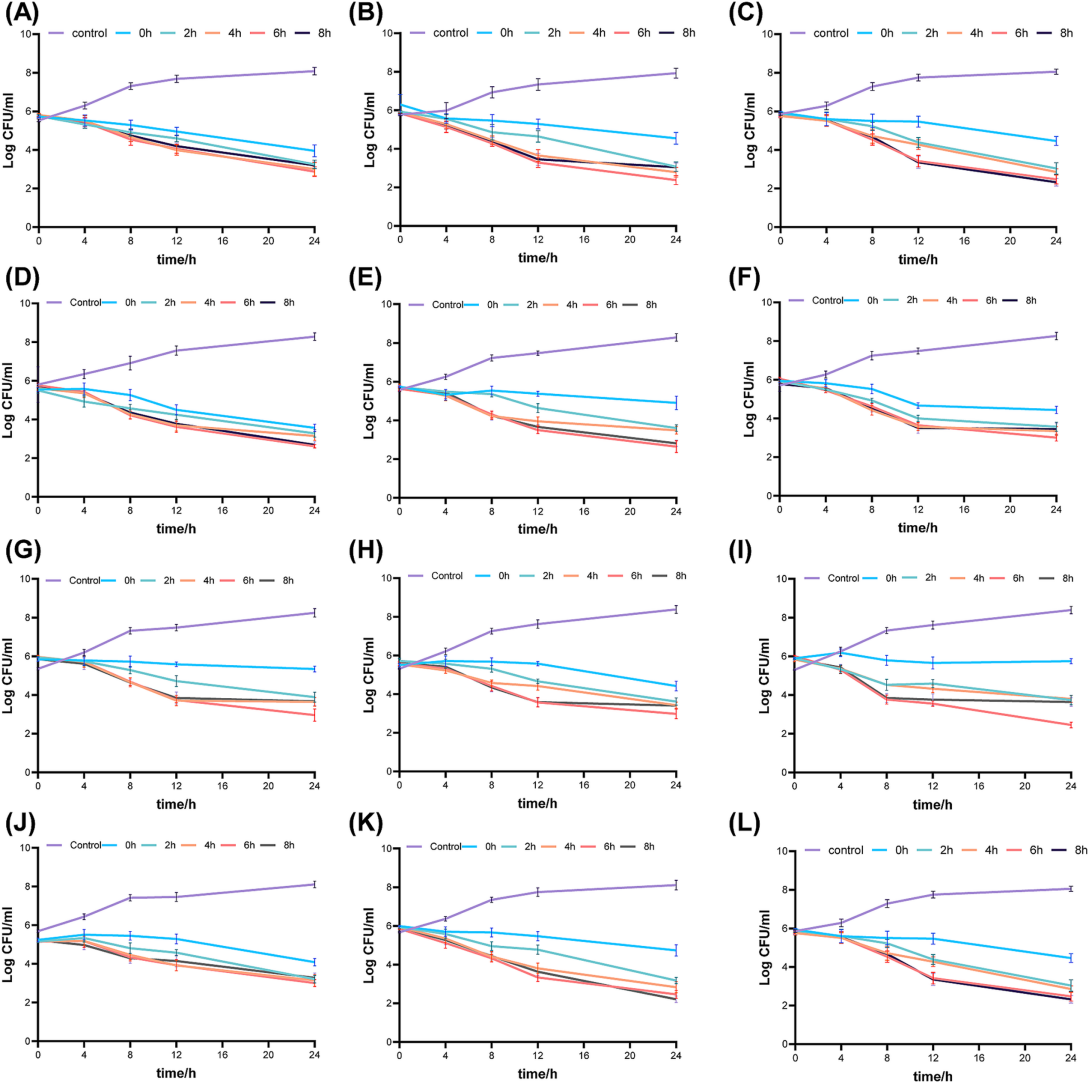
Bactericidal activity of fosfomycin and linezolid administered simultaneously or sequentially against mrsa in Static-concentration time- kill studies. Different dose, **(A)** 43300 1 × mic fos + 0.5 × mic lzd **(B)** 43300 0.5 × mic fos + 1 × mic lzd **(C)** 43300 1 0.5 × mic fos + 0.5 × mic lzd **(D)** Isolate 1 1 1 × mic fos + 0.5 × mic lzd **(E)** Isolate 1 0.5 × mic fos + 1 × mic lzd **(F)** Isolate 1 0.5 × mic fos + 0.5 × mic lzd **(G)** Isolate 2 1 × mic fos + 0.5 × mic lzd **(H)** Isolate 2 0.5 × mic fos + 1 × mic lzd **(I)** Isolate 2 0.5 × mic fos + 0.5 × mic lzd **(J)** Isolate 3 1 × mic fos + 0.5 × mic lzd **(K)** Isolate 3 0.5 × mic fos + 1 × mic lzd **(L)** Isolate 3 0.5 × mic fos + 0.5 × mic lzd. Control: no drug; 0 h, concurrent administration group; 2 h, 4 h, 6 h, 8 h, sequential administration group, interval time.

Figure 2 illustrates the pharmacodynamic activity of fosfomycin and linezolid in static bactericidal assays with both simultaneous and continuous dosing regimens. In the Concurrent administration group, the three different dose combinations resulted in a 2–3 log_10_ CFU/mL reduction in 24-h colony counts for all three clinical isolates as well as *ATCC 43300*. The bactericidal efficacy in the continuous dosing group was found to be closely dependent on the dosing interval. Compared to the concurrent dosing group, the 24-h colony count reduction was generally 0–1 log_10_ CFU/mL at a 2-h dosing interval, but increased to 1–2 log_10_ CFU/mL with extended dosing intervals of 4–8 h, with the most significant bactericidal effects observed at 4 or 6-h intervals. Overall, the bactericidal activity of continuous dosing was consistently superior to that of simultaneous dosing across all tested conditions.

### 3.3 Dynamic time-kill assay results

**Figure 3:**
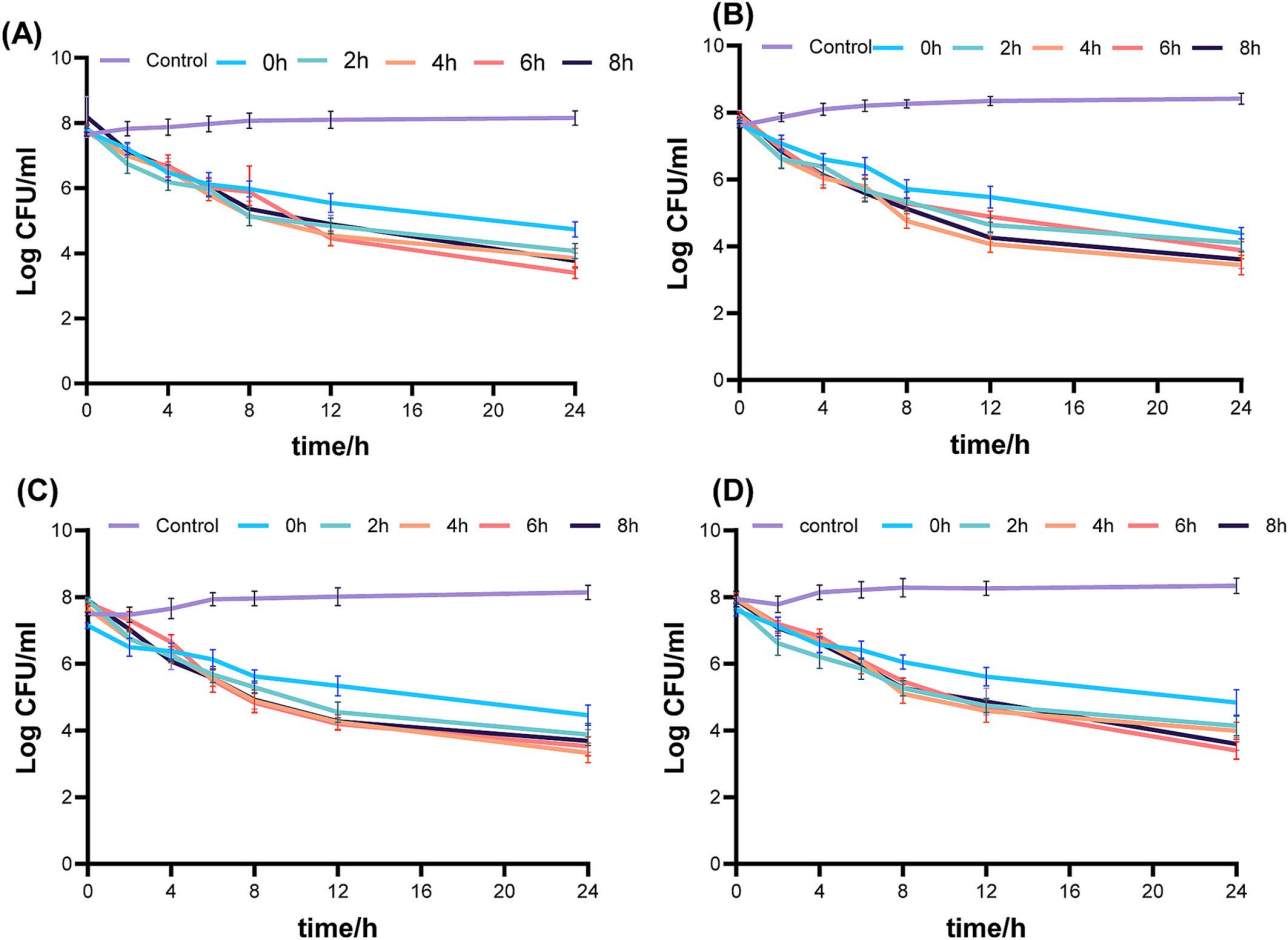
Simultaneous or sequential administration of fosfomycin and linezolid against mrsa with time-kill curves in a dynamic PK/PD model. **(A)** 43300; **(B)** Isolate 1; **(C)** Isolate 2; **(D)** Isolate 3. Control: no drug; 0 h, concurrent administration group; 2 h, 4 h, 6 h, 8 h, sequential administration group, interval time. Control: no drug; 0 h concurrent administration group; 2 h, 4 h, 6 h, 8 h, sequential administration group, interval time.

Figure 3 displays the pharmacodynamic activity of fosfomycin and linezolid administered concurrently and sequentially in an *in vitro* PK/PD simulation model. The no-treatment control group showed robust bacterial growth. In the concurrent administration group, the 24-h colony count reductions were 3.13 log_10_ CFU/mL for *ATCC 43300*, 3.79 log_10_ CFU/mL for Isolate 1, 3.54 log_10_ CFU/mL for Isolate 2, and 3.09 log_10_ CFU/mL for strain 3. Despite the administration of lower doses in the continuous dosing group, the reduction in 24-h colony counts was observed as 0–2 log10 CFU/mL for *ATCC 43300*, 0–1 log10 CFU/mL for isolate 1, 1–2 log10 CFU/mL for isolate 2, and 0–2 log10 CFU/mL for isolate 3, in comparison to the concurrent dosing group.

### 3.4 Validation of *in vitro* PK/PD model concentrations

**Figure 4:**
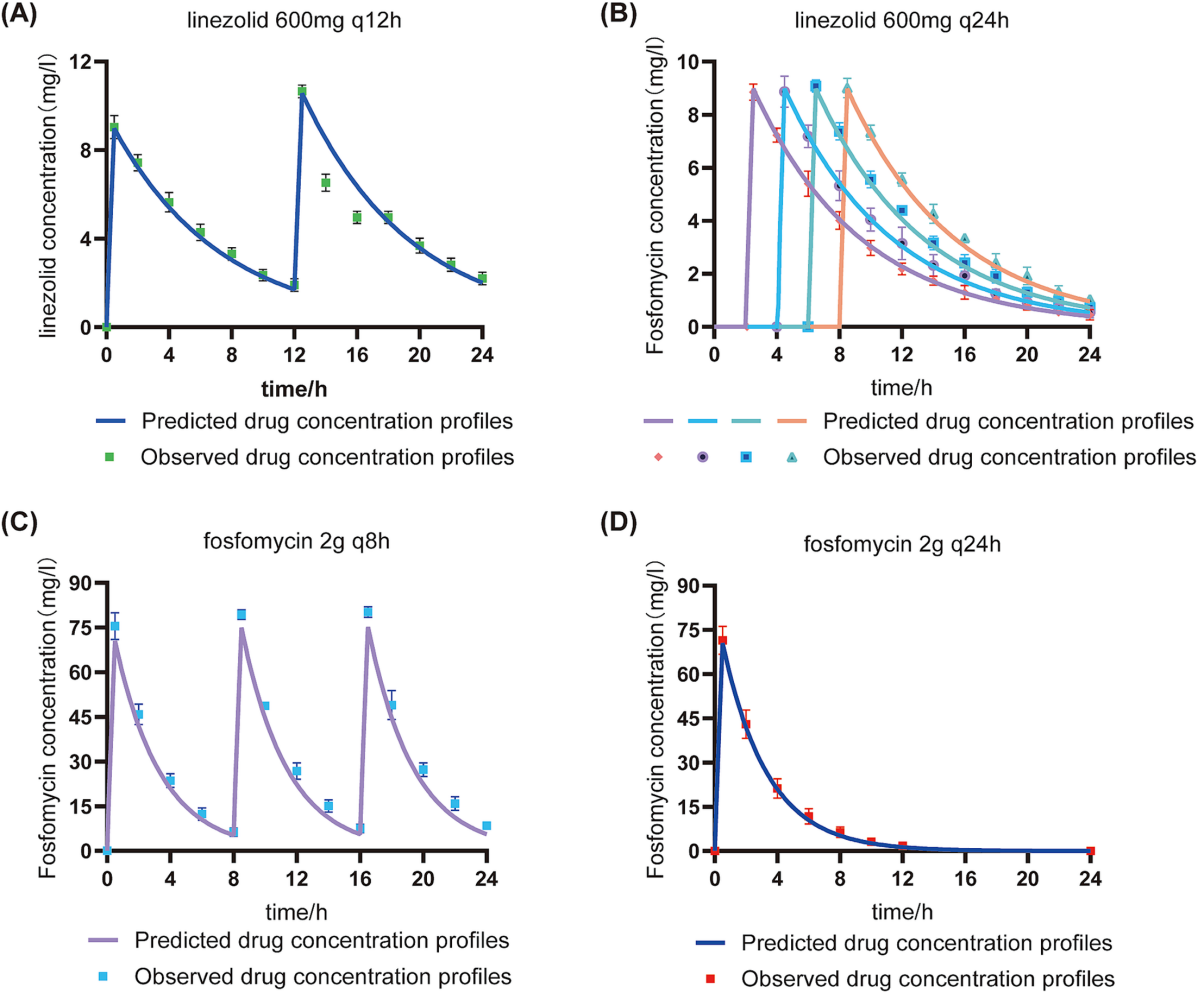
Validation of target and measured drug concentrations in the in vitro PK/PD model under sequential and concurrent administration regimens. Predicted drug concentration profiles: Pharmacokinetic curves derived from model-based simulations using initial dosage parameters.Observed drug concentration profiles: Experimentally measured drug concentrations in the central chamber.q8h: Concurrent administration group; q12h: Concurrent administration group; q24h: Sequential administration group. **(A)**: Linezolid concentration in the concomitant administration group; **(B)**: Linezolid concentration in the sequential administration group; **(C)**: Fosfomycin concentration in the concomitant administration group; **(D)**: Fosfomycin concentration in the sequential administration group.

In the in vitro PK/PD model, the concentrations of linezolid (600 mg) and fosfomycin (2 g) in the central compartment were maintained within ±30% of the target concentrations (except for the 24-h fosfomycin concentration), indicating successful establishment of the PK model (Figure 4). The pharmacokinetic parameters of fosfomycin and linezolid administered concurrently are shown in Table 2. The *f*AUC_0-24h_ of fosfomycin in the sequential administration group was approximately one-third of that in the concurrent administration group, while the *f*AUC_0-24h_ of linezolid in the sequential administration group was about half that of the concurrent administration group.

**Table 2:** Pharmacokinetic parameters validation of linezolid and fosfomycin in an in vitro PK/PD model.

		*f*AUC_0-24h_ /MIC		
Medicine	*f*AUC_0-24h_ (mg*L/h)	Isolate 1	Isolate 2	Isolate 3
LZD (600 mg)				
PK/PD model (q12h)	119.79 ± 9.03	55.38	31.11	31.03
PK/PD model (q24h)	61.43 ± 3.29	31.11	14.53	15.98
FOS (2 g)				
PK/PD model (q8h)	753.13 ± 55.77	199.84	95.33	87.17
PK/PD model (q24h)	235.31 ± 2.38	59.01	29.62	29.12

q8h, q12h, Concurrent administration group; q24h, Sequential administration group.

### 3.5 Transmission electron microscopy results

**Figure 5:**
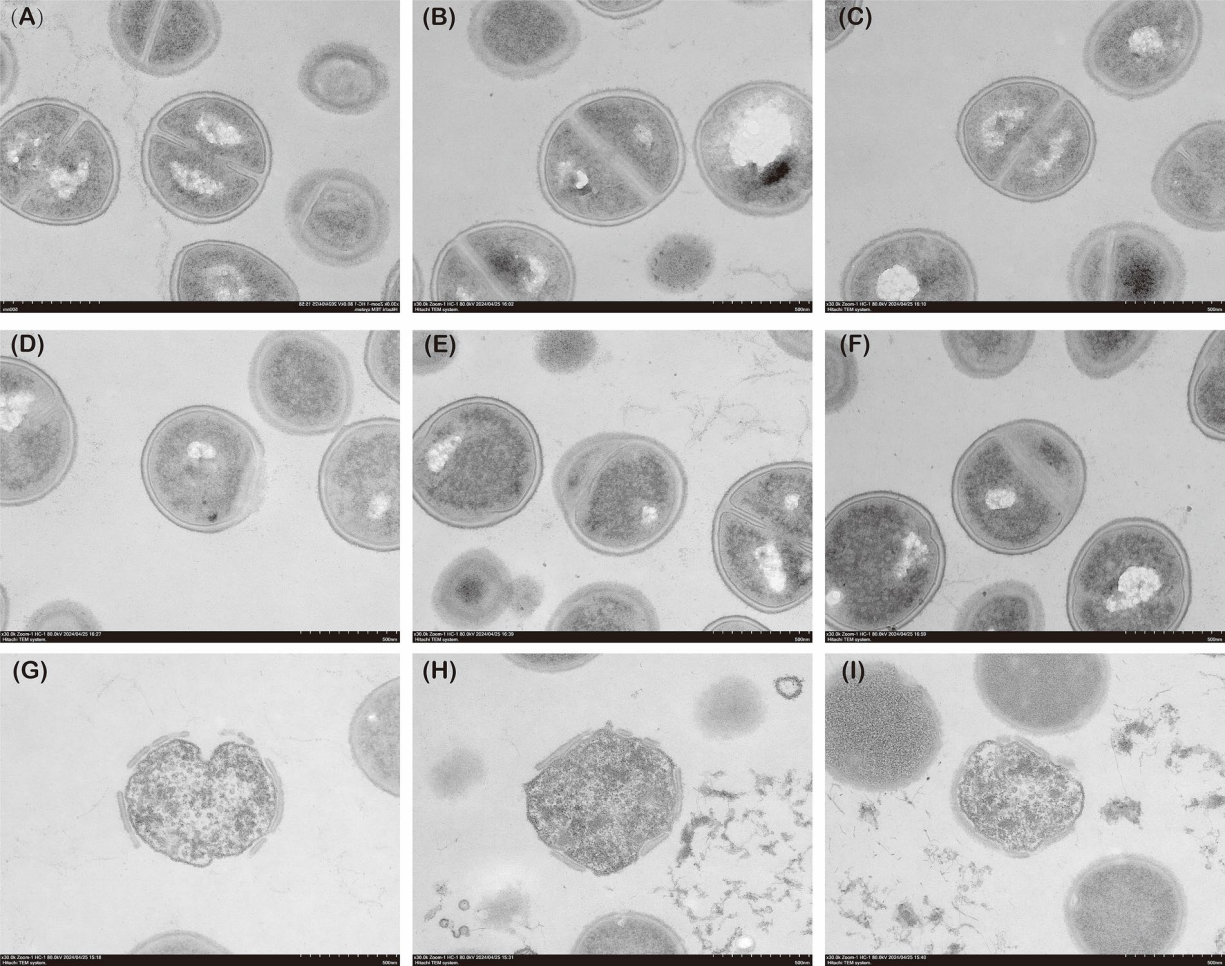
The TEM images of MRSA. **(A–C)** Control group; **(D–F)** Concurrent administration group; **(G–I)**: Sequential administration group.

Figure 5 presents the TEM analysis of *MRSA* Isolate 2 under simultaneous administration of fosfomycin (8 mg/L) and linezolid (4 mg/L) compared to sequential administration of the two drugs. In the simultaneous administration group, abnormal cell wall structures were observed. In the sequential administration group, more pronounced cell wall fragmentation and nucleoplasm loss were evident. These findings indicate that sequential administration exerts a greater destructive effect on the bacterial cell wall and nucleus compared to simultaneous administration.

## 4 Discussion

In this study, we systematically compared the *in vitro* antimicrobial efficacy of sequential and concurrent administration of fosfomycin and linezolid *against methicillin-resistant Staphylococcus aureus (MRSA)*. The results demonstrated that sequential administration was significantly more effective than simultaneous administration. In both static and dynamic bactericidal assays, the sequential administration group exhibited higher bactericidal efficiency. Transmission electron microscopy (TEM) further revealed that sequential administration caused more substantial damage to the *MRSA* cell walls and nuclei, providing new insights into optimizing therapeutic strategies for *MRSA* infections.

Previous studies have extensively explored the synergistic effects of fosfomycin and linezolid (Saravolatz and Pawlak, 2022; Tang et al., 2012; Li et al., 2020). However, most research has focused on the combined synergistic effects without considering the influence of the administration order. This study specifically addressed the differences between sequential and simultaneous administration. Through multiple concentration combination experiments, the sequential administration group consistently outperformed the concurrent group in static bactericidal assays, particularly at intervals of 4 or 6 h. The sequential administration group showed an additional reduction of approximately 1–3 log_10_ CFU/mL compared to the concurrent group, indicating a synergistic or additive effect. The bactericidal effect of different intervals also varied slightly, increasing with increasing interval time, reaching a plateau when the time was 4 h. In all dosing regimens, most of the colony counts at intervals of 8 h 24 h were less than 6 h 0.5-1log10 CFU/mL.

The effect of interval time on bacterial strains during combined antibiotic therapy is a relatively novel concept. Previous studies have investigated the impact of dosing intervals on the bactericidal efficacy of combinations involving phages and antibiotics (Mukhopadhyay et al., 2023). In this study, we observed that the bactericidal effects varied slightly with different interval times, generally increasing with longer intervals and reaching a plateau at 4 or 6 h. This pattern remained consistent even when the dosage of fosfomycin was altered, allowing us to preliminarily conclude that the fosfomycin dose does not significantly influence the efficacy of sequential administration.

Pharmacokinetic/pharmacodynamic (PK/PD) analysis is a valuable tool for evaluating antibiotic regimens. Previous studies have examined the PK/PD parameters of dosing and frequency (Mao et al., 2021; Wang et al., 2021). In this study, we used an *in vitro* PK/PD simulation model to mimic intravenous administration in humans, with the concurrent group receiving linezolid 600 mg q12h and fosfomycin 2 g q8h (Boak et al., 2014), and the sequential group receiving linezolid 600 mg q24h and fosfomycin 2 g q24h to ensure experimental feasibility. The results showed that the *f*AUC/MIC values for Isolate 1 were significantly higher than those for Isolate 2 and 3 in the concurrent administration group, consistent with previous findings that *f*AUC/MIC is positively correlated with total bacterial kill (Boak et al., 2007; VanScoy et al., 2015). At 2-h intervals, the bactericidal effect was comparable to the concurrent administration group, but at intervals of 4 h or longer, the sequential administration group showed a 1–3 log_10_ CFU/mL reduction in colony count at 24 h compared to the concurrent group. Notably, the *f*AUC and *f*AUC/MIC values were lower in the sequential administration group for both fosfomycin and linezolid. Previous reports suggest that the incidence of thrombocytopenia is 38.7% with the standard clinical dose of 600 mg of linezolid, with an even higher incidence in patients with renal insufficiency (Takahashi et al., 2011). As renal impairment increases the area under the curve (AUC), higher drug exposure can induce thrombocytopenia (Niwa et al., 2009; Crass et al., 2019). The bactericidal efficacy of linezolid and fosfomycin is directly related to the AUC/MIC ratio. Literature indicates that an AUC/MIC threshold of 100 to 119 for linezolid is effective in preventing the emergence of resistance (Rao et al., 2020). In our study, the AUC/MIC ratio for the linezolid sequential dosing group was approximately half that of the concomitantly dosed group, while the AUC/MIC for fosfomycin reached as low as one-third of the corresponding value. Despite these lower ratios, the bactericidal efficacy of the sequential dosing group remained superior to that of linezolid alone. This suggests that sequential dosing can effectively lower the AUC/MIC thresholds for both fosfomycin and linezolid, allowing for reduced drug dosages while achieving similar pharmacodynamic goals, thereby minimizing the risk of adverse effects.

Compared with the other two clinical isolates, Isolate 3 exhibited only an additive effect. In the static bactericidal assay, no significant differences were observed between Isolate 3 and the other strains, likely because the relatively short experimental duration and limited drug concentrations were insufficient to fully capture the advantages of synergism. However, in the dynamic bactericidal assay, a clear distinction emerged: when administered simultaneously, the colony count of Isolate 3 at 24 h was 0.3–0.6 log₁₀ CFU/mL higher than that of the other strains. Notably, sequential administration compensated for the differences between additive and synergistic effects by initially disrupting the bacterial cell wall, thereby enhancing overall bactericidal activity. The dynamic time-kill assay further demonstrated that the sequential dosing regimen effectively improved the antimicrobial efficacy against Isolate 3. These findings suggest that FICI values alone may not fully predict the therapeutic benefits of sequential administration.

Several studies have examined bacterial morphology under drug resistance conditions in *MRSA* (Hotz et al., 2024). Using TEM, they observed that the cell walls of multidrug-resistant *MRSA* strains were significantly thickened (Qi et al., 2019). In the present study, TEM was employed to compare the morphology of bacteria in the sequential, concurrent, and control groups. The cell wall structure in the concurrent group remained unchanged, indicating that co-administration was less prone to inducing resistance. In contrast, the sequential administration group exhibited significantly thinner and structurally compromised cell walls, with complete loss of nuclear material. These results suggest that sequential administration may exert a more destructive effect on *MRSA* than concurrent administration. This may be attributed to the mechanisms of action of the two drugs: fosfomycin inhibits bacterial cell wall synthesis by blocking the initial step involving phosphoenolpyruvate synthase (Falagas et al., 2016), while linezolid exerts its antibacterial effect by inhibiting bacterial protein synthesis (Hashemian et al., 2018),When *MRSA* is exposed to fosfomycin first, the integrity of the bacterial cell wall is disrupted, allowing linezolid to penetrate more easily and act within the bacterial cell. In conclusion, sequential administration of fosfomycin and linezolid exerts a more potent bactericidal effect on *MRSA* and is less likely to induce resistance.

*MRSA* resistance has become a significant challenge in clinical treatment (Peacock and Paterson, 2015), especially in the context of vancomycin, a first-line drug that is limited by resistance and nephrotoxicity (Chavanet, 2013). There is an urgent need to develop more effective therapeutic strategies. Linezolid, as an alternative to vancomycin for *MRSA* infections, has shown good initial antimicrobial activity; however, with increasing resistance, its efficacy as monotherapy is diminishing (Chen et al., 2020). As the problem of antibiotic overuse continues to grow (Tang et al., 2023), resistance rates are rising, and the development of new antibiotics requires substantial time and financial investment. In contrast, optimizing the use of existing antibiotics, particularly by adjusting delivery strategies such as sequential administration, offers a cost-effective means to improve patient outcomes and address drug-resistant infections.

Although this study provides preliminary evidence supporting the use of sequential administration of fosfomycin and linezolid, several limitations remain. First, the *in vitro* findings need to be validated in animal models and clinical trials to confirm the efficacy and safety of sequential administration. Second, this study was limited to three clinically isolated *MRSA* strains, which may not fully represent the diversity of MRSA strains with varying resistance profiles. Additionally, the optimal dosage and timing intervals for sequential administration were not fully explored in this study, and further research is needed to evaluate the impact of these key parameters on treatment efficacy.

In summary, this study systematically compared the antimicrobial efficacy of sequential versus concurrent dosing of fosfomycin and linezolid against MRSA. The results indicate that sequential dosing is significantly more effective than concurrent dosing, providing a new approach for optimizing MRSA treatment. Future studies should further validate and refine the sequential dosing strategy, offering new therapeutic options to address the growing challenge of antibiotic resistance.

## Data Availability

The original contributions presented in the study are included in the article/supplementary material, further inquiries can be directed to the corresponding authors.
